# Rethinking the diagnosis of double‐seronegative myasthenia gravis

**DOI:** 10.1111/ene.16151

**Published:** 2023-11-17

**Authors:** Sohyeon Kim, Mi‐Yeon Eun, Jae‐Joon Lee, Hung Youl Seok

**Affiliations:** ^1^ Department of Neurology, Dongsan Hospital Keimyung University School of Medicine Daegu Korea; ^2^ Department of Neurology, School of Medicine Kyungpook National University, Kyungpook National University Chilgok Hospital Daegu Korea

Dear Editor,

We found an interesting paper by Martinez‐Harms et al. [1] that presents a comprehensive investigation into the clinical characteristics and treatment outcomes associated with double‐seronegative myasthenia gravis (dSNMG). This specific condition is defined by the absence of antibodies targeting the acetylcholine receptor (AChR) and muscle‐specific tyrosine kinase. The findings of the study, particularly the significant improvements observed in the Myasthenia Gravis Impairment Index (MGII) and the Single Simple Question scores during the latest post‐treatment clinical evaluation of the dSNMG patients, carry substantial significance. These encouraging treatment responses have led the authors to hypothesize an immune‐based pathophysiological mechanism at the core of this disorder. However, certain aspects require further investigation and refinement.

A key concern is the potential inclusion of individuals in the dSNMG group who may not have true myasthenia gravis (MG). Based on the changes in Myasthenia Gravis Foundation of America status observed in this study, nearly half of the dSNMG patients showed resistance to conventional treatments [1]. Within this subgroup, 51.3% experienced improvement, whilst 48.7% experienced no change, worsening or exacerbation. Similarly, based on MGII changes, 59.8% showed positive responses whilst 40.2% did not. In general, the prevalence of refractory MG, which remains unchanged or worsens despite appropriate use of steroids and at least one immunosuppressant, is estimated to be 10%–20% [2]. Whilst the exact proportion of refractory MG in dSNMG patients is difficult to determine from the data presented in this study [1], the substantial range of non‐responders to treatment (approximately 40%−50%) suggests that cases resembling MG may be included in the dSNMG population. Many immunological disorders include response to immunotherapy in their diagnostic criteria, and failure to achieve positive results should prompt a re‐evaluation of the diagnosis.

The authors' main diagnostic method for dSNMG relied primarily on clinical symptoms combined with the assessment of abnormal results from repetitive nerve stimulation and/or single fiber electromyography [1]. However, this approach may inadvertently include other conditions that mimic MG, as certain conditions may present with abnormal repetitive nerve stimulation and single fiber electromyography findings similar to those observed in MG [3]. Notably, the neostigmine test response was not included in the diagnostic process for MG. As the authors themselves point out, patients diagnosed with dSNMG should be thoroughly evaluated for possible alternative diagnoses such as other myasthenic syndromes and various neuromuscular disorders, especially if they do not respond to treatment.

The authors did not investigate other MG autoantibodies such as low‐density lipoprotein receptor‐related protein 4, titin, ryanodine receptor, agrin and cortactin. For improved diagnostic accuracy, a comprehensive analysis of additional MG autoantibodies may be required to differentiate true dSNMG patients [4, 5].

In conclusion, the study by Martinez‐Harms et al. significantly advances our understanding of the clinical aspects and treatment outcomes of dSNMG. As highlighted by the authors, authentic dSNMG is considered to be part of the same MG spectrum as AChR‐ antibody‐positive MG, with comparable clinical features and a positive response to conventional immunotherapy. However, the reliability of this contribution depends on an accurate diagnosis within this cohort.

## CONFLICT OF INTEREST STATEMENT

None.

## Data Availability

Data sharing is not applicable to this article as no new data were created or analyzed in this study.
